# Hybrid coexpression link similarity graph clustering for mining biological modules from multiple gene expression datasets

**DOI:** 10.1186/1756-0381-7-16

**Published:** 2014-08-18

**Authors:** Saeed Salem, Cagri Ozcaglar

**Affiliations:** 1 Department of Computer Science, North Dakota State University, Fargo, ND 58102, USA; 2 Amazon, Seattle, WA 98101, USA

**Keywords:** Coexpression, Biological networks, Mining, Frequent subnetworks, Hybrid similarity

## Abstract

**Background:**

Advances in genomic technologies have enabled the accumulation of vast amount of genomic data, including gene expression data for multiple species under various biological and environmental conditions. Integration of these gene expression datasets is a promising strategy to alleviate the challenges of protein functional annotation and biological module discovery based on a single gene expression data, which suffers from spurious coexpression.

**Results:**

We propose a joint mining algorithm that constructs a weighted hybrid similarity graph whose nodes are the coexpression links. The weight of an edge between two coexpression links in this hybrid graph is a linear combination of the topological similarities and co-appearance similarities of the corresponding two coexpression links. Clustering the weighted hybrid similarity graph yields recurrent coexpression link clusters (modules). Experimental results on Human gene expression datasets show that the reported modules are functionally homogeneous as evident by their enrichment with biological process GO terms and KEGG pathways.

## Background

Advances in high throughput technologies have enabled scientists to accumulate vast amounts of genomic data, e.g. mRNA expression for different organisms under diverse biological and environmental conditions, and protein-protein interaction networks. Integrative analysis of multiple omic datasets (e.g., interaction networks and gene expression) has the potential to elucidate the intricate interactions involved in biological processes, and has been employed for functional annotation [1], active module discovery [2], and biomarker discovery [3].

Functional annotation and biological inference based on a single gene expression dataset has limitations due to experimental noise [4]. Recently, research has focused on integrating multiple gene expression datasets to strengthen the evidence of coexpression patterns. Genes that show correlated expression profiles in multiple experiments have been proposed for module discovery and functional annotation [1,4].

Most of the existing approaches for mining multiple expression datasets represent the datasets as gene coexpression networks and mine these networks for interesting gene sets (modules) [4,5]. In these graph-theoretic approaches, each dataset is represented as a coexpression network whose nodes represent the genes and links represent significant correlation between genes. Existing algorithms for mining significant patterns from coexpression networks mainly follow network clustering, pattern enumeration approaches, or a combination of both.

Network clustering based algorithms work on an aggregate graph constructed from multiple conexpression networks. Lee et al. [1] proposed an approach that builds an unweighted summary graph that only has edges which occur in at least a minimum number of graphs. The MCODE algorithm [6] for network clustering is employed on the aggregate summary graph to extract highly connected genes (modules). Experiments showed that coexpression patterns mined from multiple independent microarray datasets are more likely to be functionally relevant and thus improve gene function predictions [1].

Clustering the aggregate graph results in false positive modules since the links between the edges in a given module can be scattered across the graphs but appear together in the aggregate graph. To overcome these false positive modules, Hu et al. [4] proposed the CODENSE algorithm, a two-step approach for mining coherent dense subgraphs. In the first phase, dense modules are extracted from the aggregate graph. The second phase uses the edge occurrence similarity and partition these dense modules into smaller modules whose edges show high edge occurrence similarity. Depending on the similarity threshold employed in the second phase, a false-positive module extracted from the first phase can be split into edge sets that do not appear frequently together in the graphs.

The other category of methods for mining multiple coexpression networks follow a pattern-mining approach. Huang et al. [7] proposed an algorithm that reverses the order of the two steps in CODENSE for integrating multiple coexpression graphs. First, a binary matrix, whose rows correspond to edges of the template graph, and columns correspond to the graphs, is constructed. The value of each entry represents the presence/absence of the edge in the corresponding graph. Biclusters are extracted from this binary edge matrix using a simulated annealing-based approach. A bicluster in this case is a set of edges that have similar occurrences in a subset of the graphs. Each bicluster induces a subgraph from the template graph and then connected components are extracted from this subgraph. We proposed the MFMS algorithm [8] that first mines maximal frequent edge sets. For each maximal frequent edgeset, a collection of highly-connected subgraphs (*k*-cliques and percolated *k*-cliques) is extracted from the induced subgraph within the template graph.

Frequent subgraph mining algorithms can be employed to discover frequent coexpression subnetworks. Since the coexpression networks have unique labels on nodes (genes), several efficient algorithms for frequent subgraph pattern mining have been developed for this type of graphs [9-13]. Frequent subgraph mining algorithms report a large number of overlapping patterns. Therefore, Yan et. al [10] proposed an algorithm for mining a representative set of frequent patterns that meet connectivity constraints. In gene expression analysis, the same coexpression patterns might not appear in a significant number of coexpression networks and thus sets of genes that show different coexpression relationships in a number of graphs are of interest. Li et al. [5] proposed a multi-stage relaxation method (NetsTensor) on the tensor representation of multiple graphs, and found recurrent heavy subgraphs representing functional modules, transcriptional modules, and protein complexes. The NetsTensor approach is computationally intensive and takes days to finish on the data sets we are dealing with in this work.

In this paper, instead of clustering the summary graph and then extracting the highly co-occurring edge sets as in CODENSE [4] or mining biclusters then finding (highly)-connected components ([7,8]), we propose an algorithm for joint mining of modules from multiple gene expression datasets. First, we use the topological and co-occurrence similarity between coexpression links to construct a weighted graph of coexpression links. Then, we extract edge clusters from the weighted link graph. Since we cluster the edges rather than nodes, the reported modules can overlap, which is an essential feature for module discovery methods.

## Methods

A multi-layered graph is a set of graphs defined over the same set of nodes with different sets of edges. The set of graphs composing a multi-layered graph can be summarized in one graph with edge attributes. Next, we define multi-layered and edge-attributed graphs and show the representation of a multi-layered graph as an edge-attributed graph.

### **Definition1**

**Figure 1:**
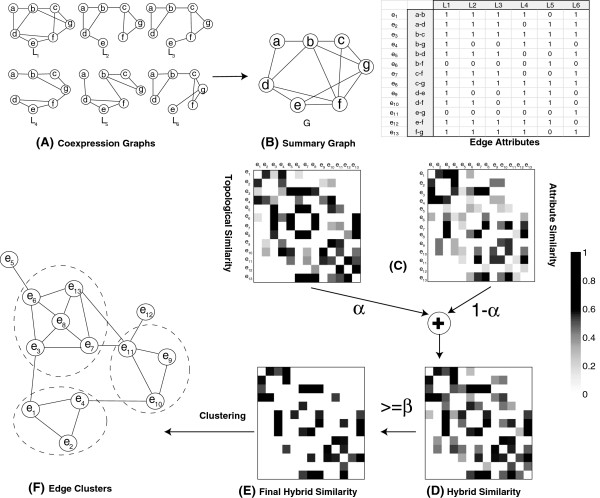
**Overview of the proposed approach(A)** Gene Expression datasets are represented as coexpression graphs; In **(B)** multiple coexpression graphs from **(A)** are represented as an edge-attributed summary graph. The topological and attribute edge similarity matrices are depicted in **(C)**, the hybrid similarity matrix is shown in **(D)** and the final hybrid similarity matrix after applying a cutoff is shown in **(E)**. The weighted hybrid graph is shown in **(F)** with the edge clusters enclosed by ovals in dotted lines.

A **multi-layered graph**$G=\{G_{1},G_{2},\ldots ,G_{d}\}$, is a set of *d* graphs such that graph *G*_*i*_ = (*V*, *E*_*i*_) for all 1 ≤ *i* ≤ *d*, where *E*_*i*_ ⊆ *V* × *V*, and *V* is the set of vertices shared by all the graphs. Figure 1(A) shows an illustrative example of a multi-layered graph with six graph layers defined over a set of seven vertices, i.e., *V* = {*a*, *b*, *c*, *d*, *e*, *f*, *g*}. Next, we define edge-attributed graphs.

### **Definition2**.

An **edge-attributed graph**$G_{e}=(V,E,L)$ consists of a set of vertices *V* = {*v*_1_, *v*_2_, ⋯, *v*_*n*_}, a set of edges *E* = {*e*_1_, *e*_2_, ⋯, *e*_*m*_}, *E* ⊆ *V* × *V*, and a function $L:E\to R^{d}$ that assigns each edge a *d*-dimensional attribute profile. Alternatively, an edge-attributed graph can be defined as *G*_*e*_ = (*V*, *E*, *X*), where $X\in R^{|E|\times d}$ is the edge attribute matrix, where *x*_*ij*_ is a binary indicator of whether edge *e*_*i*_ appears in graph *G*_*j*_.

We model a multi-layered graph, G, by an edge-attributed summary graph, *G*_*S*_ = (*V*, *E*_*S*_, *X*). The set of edges of the summary graph is the union of all the edges that appear in the multi-layered graph, G. This set of edges is represented as $E_{S}=\overset{d}{\underset{i=1}{⋃}}E_{i}$, where *E*_*i*_ is the set of edges in $G_{i}\in G$. The edge attribute matrix, *X*, captures the occurrence of edges in the graph layers, where the *i*^*t* *h*^ row in the matrix represents the presence/absence of the *i*^*t* *h*^ edge.

An Edge-attributed graph can model complex relations in multi-relational, heterogeneous networks represented as multi-layered graphs. Figure 1(A) shows an illustrative example of a multi-layered graph with six layers. The edge-attributed summary graph is shown in Figure 1(B), where the summary graph is the union of the edges that appear in the graphs shown in Figure 1(A). Edge occurrence in the graph layers is represented as an edge-attribute matrix in which the *i*^*t* *h*^ row corresponds to the *i*^*t* *h*^ edge occurrence vector. In Figure 1(B), the edge-attribute matrix has six dimensions, each of which represents a graph layer. In this work, we focus our attention on undirected, unweighted graphs. Therefore, the edge-attribute matrix representing the edge occurrences in a multi-layered graph is a binary matrix. This approach is also applicable to weighted graphs.

### Algorithm

We propose a two-step algorithm for joint mining of multi-layered graphs. First, we build a hybrid similarity matrix that is a weighted combination of the topological similarity and edge attribute similarity using the summary graph and the associated edge-attributed matrix. Once we construct the hybrid similarity matrix, we can run traditional graph clustering algorithms, e.g., spectral [14], and Markov Chain [15], on the weighted graph representing the hybrid similarity matrix to extract clusters of edges.

### Topological similarity

The algorithm constructs the topological similarity matrix, $S_{t}\in R^{\left|E|\times |E\right|}$ that captures the similarity between edges considering the structure of the summary graph. The topological similarity between edges indicates a sense of community structure that the two edges belong to. The topological similarity between two edges, *e*_*ik*_ = (*v*_*i*_, *v*_*k*_), *e*_*jk*_ = (*v*_*j*_, *v*_*k*_), sharing a common node, *v*_*k*_, is defined in terms of the common neighbors of the different nodes, as proposed in [16]:

$$S_{t}(e_{\text{ik}},e_{\text{jk}})=\frac{\left|n_{+}(i)\cap n_{+}(\;j)\right|}{\left|n_{+}(i)\cup n_{+}(\;j)\right|}$$

where *n*_+_(*i*) denotes the set of neighbors of node *i*, including the node itself. For example, the topological similarity between the two edges (*a*, *b*) and (*a*, *d*) in Figure 1(B), is 4/7, since nodes *b* and *d* share four common neighbors. The motivation behind topological similarity is that topologically similar edges are more likely to belong to the same community.

### Attribute similarity

The second edge similarity metric we employ is the attribute similarity. We construct the attribute similarity matrix whose entries measure how similar the edges are in the attribute space. For edge attributes with real values, we can employ traditional vector similarity measures such as cosine similarity, correlation coefficient, and Euclidean distance. Since the coexpression graphs are unweighted, we define the attribute similarity between two edges as the Jaccard similarity coefficient between the edges’ occurrences:

$$S_{a}(e_{\text{ik}},e_{\text{jk}})=\frac{\left|\text{gid}(e_{\text{ik}})\cap \text{gid}(e_{\text{jk}})\right|}{\left|\text{gid}(g_{\text{ik}})\cup \text{gid}(g_{\text{jk}})\right|}$$

where *gid*(*e*_*ik*_) is the set of graph identifiers in which edge *e*_*ik*_ appears. For unweighted multi-layered graphs, the attribute similarity between two edges is essentially the ratio of the number of graphs in which the two edges coexist to the number of graphs in which any of the two edges appears. Edges that are distant in the graph structure can still have attribute similarity. To avoid incorporating such attribute similarity, we only calculate the edge attribute similarity for edges that share an endpoint, i.e., edges that have non-zero topological similarity. For the data shown in Figure 1(B), *S*_*a*_((*a*, *b*), (*a*, *d*)) = 4 / 6 = 0.67, since the two edges occur together in four graphs and at least one of them appears in all six graph layers.

### Hybrid similarity

We construct the hybrid similarity matrix which combines the topological and attribute similarity between edges. Combining similarities from various sources have been proposed for clustering graphs with node attributes [17-19]. We propose a weighted combination of the topological and attribute edge similarity in a way that is similar to the hybrid metric proposed by [18] for combining node attribute and link information.

The hybrid similarity measure is a weighted combination of the topological and attribute edge similarity measures:

$$S(e_{\text{ik}},e_{\text{jk}})=\alpha \times S_{t}(e_{\text{ik}},e_{\text{jk}})+(1-\alpha )\times S_{a}(e_{\text{ik}},e_{\text{jk}})$$

where *α* is a user-specified parameter controlling the contribution of topological similarity to the hybrid similarity. The hybrid similarity matrix can be written in terms of both topological and attribute similarity matrices as follow:

$$S=\alpha \times S_{t}+(1-\alpha )\times S_{a}$$

where, *S*_*t*_ represents topological similarity, and *S*_*a*_ represents attribute similarity. The hybrid similarity matrix can be represented as a weighted graph whose nodes represent the edges in the summary graph and the weight between two nodes corresponds to the hybrid similarity between the corresponding two edges. Two edges that appear simultaneously in a small number of graphs but appear separately in many graphs will have a small attribute similarity and will be included in the hybrid similarity graph. To remove the impact of these small hybrid similarities, we apply a threshold on the hybrid similarity graph so that two edges are considered similar if the similarity exceeds a user-specified threshold, *β*. For the example in Figure 1, the hybrid similarity matrix (*α* = 0.5) is depicted in (D) and the final hybrid similarity is shown in (F) by retaining only similarities of at least 0.5 (*β*=0.5). The final weighted hybrid graph is shown in (E) with the edge clusters. 

The proposed algorithm is described in Algorithm 1. The algorithm first constructs the summary graph and the edge attribute matrix from the multi-layered graph (line 1). Then it computes the topological and attribute similarity matrices (lines 2, 3). Next, the two matrices are joined to construct the hybrid similarity matrix. The final hybrid graph is built from the hybrid similarities by removing all links with similarity values less than a threshold, *β*. In the final step, we run Markov clustering algorithm [15] on the weighted hybrid graph to extract edge clusters. For each edge cluster, the vertices in the edge-induced subgraph constitute a module. Since we cluster edges rather than nodes, the reported modules can overlap, which is essential for understanding the structure in biological interactions.

### Datasets

The gene expression datasets used in the experiments are selected from the 65 datasets used in [7]. We also used the same datasets in [8]. Among the 65 gene expression datasets, 52 are Affymetrix microarray datasets, and the remaining 13 are cDNA expression datasets. There are 3397 genes that appear only in the cDNA datasets. Therefore, to avoid creating a sparse data, we only constructed the coexpression graphs for the 52 Affymetrix microarray datasets. The correlation between the expression profiles in a gene expression dataset is modeled as a coexpression graph in which nodes represent genes and an unweighted edge between two genes indicates significant correlation between the two genes’ expression profiles. The strength of the correlation is measured by the Pearson’s correlation between the two genes’ expression profiles. To ensure that the observed correlation is significant, we employ a cut-off of 0.01 on the *p*-value of the correlation. No edge is added to the coexpression graph between two genes if the correlation, between their expression profiles, is not significant (*p*-value >0.01), regardless of how strong the correlation is.

We constructed the summary graph and the edge attribute matrix for these 52 coexpression graphs. The number of all edges in the summary graph is very large (49,817,037). The number of graphs in which edges occur (edge frequency) vary between 1 and 33, with more than 38 million edges appearing in at most 2 graphs. Edges that appear in a very small number of graphs will have low co-occurrence similarity with frequent edges and retaining these not-so-frequent edges will lead to a large summary graph and a very sparse edge occurrence matrix. Therefore, we prune the edges that occur in a small number of graphs. For example, if we keep the frequent edges that appear in at least 7 graphs, we get a summary graph with with 9,784 nodes and 308,162 edges. Further increasing the frequency threshold to 10 graphs leads to a summary graph with 4,133 nodes and 32,741 edges. We used a minimum edge frequency of 10 to generate the edge-attributed graph throughout the experiments.

## Results

In order to assess the effectiveness of the proposed algorithm on extracting biological modules from multiple gene coexpression graphs, we performed an experimental evaluation of the algorithm using multiple human gene expression datasets. Furthermore, we analyzed some topological characteristics of the reported clusters in terms of size, density and recurrence. We have also performed enrichment analysis to assess the functional homogeneity of the extracted modules. The algorithm for constructing the hybrid similarity graph was implemented in C++ and all experiments were run on a 2.75Ghz Machine running Linux Ubuntu with 8GB Memory. We used the Markov clustering algorithm implementation that is provided by the authors of [15].

### Topological properties of edge clusters

In order to find edge clusters, we use the Markov Chain Clustering (MCL) algorithm [15] on the weighted hybrid similarity graph. We have chosen MCL since it has been shown to report meaningful clusters in biological networks [20]. The inflation parameter for the MCL algorithm is kept at 2.0 throughout the experiments.

To investigate the topological characteristics of edge clusters in terms of their density and frequency in multi-layered graphs, we analyzed the topological properties of the edge clusters extracted by the MCL algorithm from the hybrid similarity graph. We ran experiments for varying the minimum hybrid similarity thresholds, *β*∈[ 0.0,1], that control the size of the hybrid similarity graph, |*E*_*S*_|, by retaining only highly similar edges. A clustering of the hybrid similarity graph produces a partition of the graph nodes which represent edges of the original summary graph. Formally, a clustering *M* of the hybrid graph is a set of mutually exclusive edge sets:

$$M=\{{\text{EC}}_{1},{\text{EC}}_{2},\cdots \;,{\text{EC}}_{\left|M\right|}\}$$

where *EC*_*i*_ is a set of nodes from the hybrid similarity graph. Recall that the nodes in the hybrid similarity graph represent the edges of the summary graph. We only consider clusters that have at least 4 nodes (originally edges). Choosing 4 edges in the summary graph ensures that each module has at least 4 genes.

For an edge cluster *EC*, the edge-induced subgraph from the summary graph is denoted as *G*_*S*_[*EC*]. Let *V*(*EC*) denote the set of vertices in the edge-induced summary subgraph, i.e., *V*(*EC*) = *V*(*G*_*S*_[*EC*]). The average number of vertices and edges in the edge clusters are denoted as $\bar{V}$ and $\bar{E}$, respectively. Table 1 shows the topological properties of the reported patterns for varying hybrid similarity thresholds, *β*, that control the size of the hybrid similarity graph, |*E*_*S*_|, i.e., the number of edges. These topological properties are reported for *α* = 0.5 which allows for the topological and attribute similarity to contribute equally to the hybrid similarity measure.

**Table 1:** **Topological analysis of edge clusters for varying** ***β***

		**Edge clusters**					**0.5-sup**		**0.75-sup**		**1.0-sup**	
***β***	**|** ***E***_***S***_**|**	**|** ***M*** **|**	$\bar{V}$	$\bar{E}$	$\bar{\text{RE}}$	$\bar{\rho }$	$\bar{\text{sup}}$	**|** ***M***^***′***^**|(*%*)**	$\bar{\text{sup}}$	**|** ***M***^***′***^**|(*%*)**	$\bar{\text{sup}}$	**|** ***M***^***′***^**|(*%*)**
0	2273510	421	48.5	77.2	0.74	0.16	6.8	337(80)	1.9	46(11)	0.3	0(0)
0.1	1945908	470	44.3	69.1	0.74	0.16	6.9	382(81)	2	57(12)	0.3	0(0)
0.2	873741	1559	15.8	19	0.58	0.25	9.1	1546(99)	4	578(37)	1.1	11(1)
0.3	226832	1777	10.5	10.4	0.41	0.28	10.3	1777(100)	5.9	1380(78)	2.4	209(12)
0.4	55897	896	8.5	8.2	0.36	0.31	11.5	896(100)	7.8	880(98)	4.1	383(43)
0.5	15779	276	9.5	9.4	0.32	0.3	11.7	276(100)	8.9	275(100)	5.4	162(59)
0.6	3864	147	7.7	7.4	0.33	0.33	12.6	147(100)	10	147(100)	6.9	127(86)
0.7	625	25	6.2	6.8	0.45	0.45	15.1	25(100)	12	25(100)	9.1	24(96)
0.8	625	4	5.2	6.5	0.66	0.66	19	4(100)	14.2	4(100)	10.5	4(100)
0.9	20	1	4	6	1	1	23	1(100)	22	1(100)	20	1(100)

An *α* value of 0.5 is used and only edge clusters with at least 4 edges are retained.

The set of edges in an edge cluster often constitute only a subset of the entire set of edges that are present in the summary graph between the same set of vertices. An edge whose occurrence does not significantly overlap with the occurrences of the edges in the edge cluster will not be reported as part of the edge cluster. Moreover, edges that appear in a small number of graphs will not be in the edge cluster as their similarities with frequent edges can be below the *β* threshold. The set of edges that appear among the set of vertices of the edge cluster are essentially the edges in the induced summary graph based on these set of vertices, i.e., *E*(*G*_*S*_[ *V*(*EC*)]). We compute the ratio (*RE*) of the edges in the edge cluster to the total number of edges present between the vertices in the summary graph as follows:

$$\text{RE}({\text{EC}}_{i})=\frac{\left|{\text{EC}}_{i}\right|}{\left|E(G_{S}[\;V({\text{EC}}_{i})]\;)\right|}$$

In the topological analysis, we report the average edge ratio for all edge clusters, $\bar{\text{RE}}$. Moreover, we compute the density of the edge-induced subgraph for each edge cluster:

$$\rho ({\text{EC}}_{i})=\frac{2*|{\text{EC}}_{i}|}{\left(|V({\text{EC}}_{i})|\times (|V({\text{EC}}_{i})|-1)\right)}$$

We report the average density of the edge-induced subgraphs of the reported edge clusters, $\bar{\rho }$. We can see the impact of varying the minimum similarity threshold (*β*) on these structural measures. For small *β* threshold (0,0.1), the number of edges in the summary graph is very large, thus resulting in dense hybrid graphs with densities of 0.26 and 0.22, respectively. For these dense hybrid graphs, the MCL reports large edge clusters with an average size higher than 40 nodes ($\bar{V}$) and around 70 edges ($\bar{E}$). At minimum hybrid similarity, *β*, threshold of 0.3, the algorithm reports medium-sized clusters with average number of nodes equal to 10.

As we increase *β* and thus reduce the number of edges in the hybrid graph (also the density), the average number of nodes and edges in the edge clusters decrease. This is expected since as we increase *β*, we require edges to have significant similarities and thus smaller number of edge pairs qualify as links in the hybrid graph. In line with this observation, we see that for small *β* thresholds, the edge clusters are large and contain most of the edges in the induced summary graph: for *β*=0.1, the average ratio of edges in the edge clusters to the edges in the induced summary graph is relatively high, $\bar{\text{RE}}=0.74$. As we increase *β*, the average ratio of edges in the edge clusters decreases since fewer edges qualify to be in the hybrid graph.

### Edge cluster recurrence

The next two topological measures we compute for the edge clusters are aimed at measuring how recurrent these edge clusters are in the multiple coexpression graphs. Since the similarity measure between edges is not transitive, all edges in an edge cluster are not pairwise similar and thus are not expected to appear together in the same set of graphs. The reported edge patterns are not exact frequent subgraph patterns (sets of edges), that is, not all of the reported edges in an edge cluster will appear in several graphs. Therefore, we measure the *γ*-approximate occurrence of edge clusters. An edge cluster, *EC*, has a *γ*-approximate occurrence in a graph if at least ⌈*γ*∗|*EC*|⌉ edges of the edge cluster appear in the graph. The *γ*-support of an edge cluster is the number of graphs in which the edge cluster has *γ*-approximate occurrence.

$$\gamma -\text{sup}({\text{EC}}_{i})=|\{G_{i}\;|\;|{\text{EC}}_{i}\cap E(G_{i})|\geq \gamma *|{\text{EC}}_{i}|\}|$$

For *γ* = 1, the *γ*-support of a set of edges is equivalent to the conventional support employed in the frequent pattern mining literature. In Table 1, we report the average approximate support (*γ*-*sup*) for all edge clusters, denoted as $\bar{\text{sup}}$. Moreover, we report the number of edge clusters that have approximate support (*γ*-*sup*) greater than *minsup* graphs (*minsup* = 5), i.e., have *γ*-approximatecvaph layers. We also report the percentage of edge clusters with approximate support (*γ*-*sup*) greater than 5 graphs (in parentheses). We investigated the effect of minimum hybrid edge similarity (*β*) on *γ*-support of the reported patterns. For small *β* thresholds ([0,0.3]), the majority of the reported edge clusters are not truly recurrent edges as evident by the small percentage of the reported edge clusters that appear in at least 5 graph layers (see the last column in Table 1). As we increase *β*, the reported edge clusters become more recurrent. For *β* ≥ 0.6, a large percentage of the edge clusters (≥ 80*%*) are recurrent in at least 5 graphs.

The topological characteristics of the edge clusters for *α* = 0 are shown in Table 2. An *α* value of 0.0 means that the hybrid similarity is entirely based on the attribute similarities between edges that share common nodes and topological similarity between edges is not incorporated in the hybrid similarity. We observed similar trends to the ones in Table 1 for *α* = 0.5. However, for *α* = 0 we have to use a high hybrid similarity threshold to obtain recurrent edge clusters similar to the ones observed for *α* = 0.5. For example (*β* = 0.6), while for *α* = 0.5, 86*%* of the reported edge clusters are frequent in at least 5 graphs, the percentage drops to 70*%* for *α* = 0.

**Table 2:** Topological analysis of edge clusters reported for hybrid similarity graphs

		**Edge clusters**					**0.5-sup**		**0.75-sup**		**1.0-sup**	
***β***	**|** ***E***_***S***_**|**	**|** ***M*** **|**	$\bar{V}$	$\bar{E}$	$\bar{\text{RE}}$	$\bar{\rho }$	$\bar{\text{sup}}$	**|** ***M***^***′***^**|(*%*)**	$\bar{\text{sup}}$	**|** ***M***^***′***^**|(*%*)**	$\bar{\text{sup}}$	**|** ***M***^***′***^**|(*%*)**
0	2273510	436	52.1	74.6	0.72	0.15	6.7	356(82)	1.9	49(11)	0.3	2(0)
0.1	2197627	442	51.8	73.6	0.71	0.15	6.8	360(81)	1.9	48(11)	0.3	2(0)
0.2	1678507	522	45.9	61.9	0.68	0.16	7.2	460(88)	2.2	66(13)	0.4	3(1)
0.3	892412	1160	22.4	26.3	0.66	0.23	8.9	1149(99)	3.7	382(33)	0.9	6(1)
0.4	376661	2117	10.7	10.7	0.62	0.29	10.3	2117(100)	5.7	1527(72)	2.1	156(7)
0.5	128140	1974	7.7	7.3	0.57	0.33	11	1974(100)	7.3	1897(96)	3.5	568(29)
0.6	30972	939	6.9	6.4	0.52	0.35	11.7	939(100)	8.9	939(100)	5.3	661(70)
0.7	5399	226	6.6	6.1	0.46	0.37	12.5	226(100)	10.6	226(100)	7.7	215(95)
0.8	1243	69	6.1	5.5	0.43	0.39	12.7	69(100)	11.5	69(100)	9.6	69(100)
0.9	171	7	5.4	4.6	0.43	0.4	12.4	7(100)	12.1	7(100)	11	7(100)

An *α* value of 0.0 is used and only clusters with at least 4 edges are analyzed.

### Impact of topological similarity (varying *α*)

**Figure 2:**
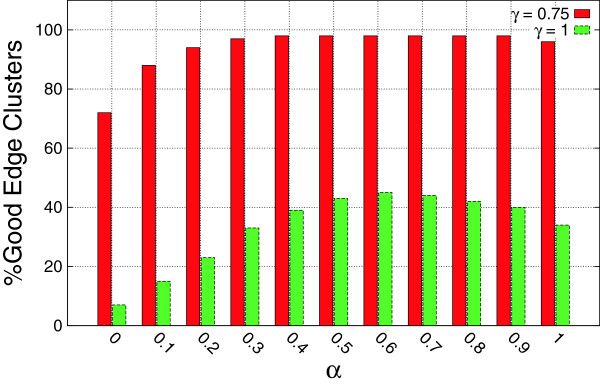
**Impact of topological similarity on the percentage of approximate frequent subgraphs.** The percentage of edge clusters that have *γ*-occurrence in at least 5 graphs is plotted for varying *α* thresholds.

**Figure 3:**
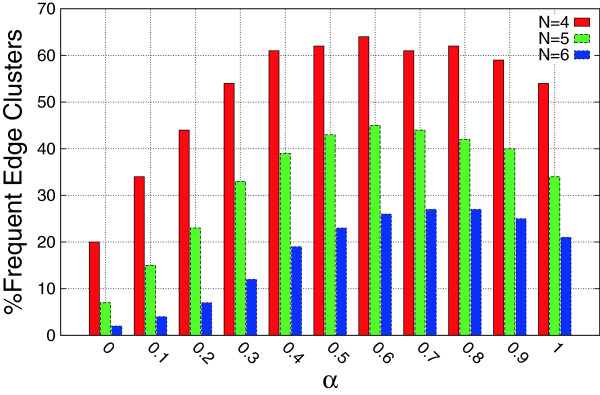
**Frequent edge clusters vs. topological similarity contribution.** The Percentage of edge clusters that appear in at least *N* graphs is shown for varying *α* thresholds. The hybrid similarity threshold, *β*, was set to 0.4.

To investigate the effect of the contribution of topological similarity on the characteristics of the reported edge clusters, we fixed *β* to 0.4 and ran the algorithm for varying *α* thresholds. Figure 2 shows the percentage of *γ*-approximate edge clusters that appear in at least 5 graphs for different *α* thresholds. Recall that for *γ* = 1, all the edges of an edge cluster must appear in a given graph to count as an occurrence. The highest percentage of edge clusters is achieved for almost balanced contribution of topological and attribute similarity. Figure 3 shows the percentage of the frequent edge clusters that appear in at least *N* graphs. For *α* values of 0.6 and 0.7, higher percentages of the edge clusters are frequent in at least *N* graphs.

### Biological significance

**Figure 4:**
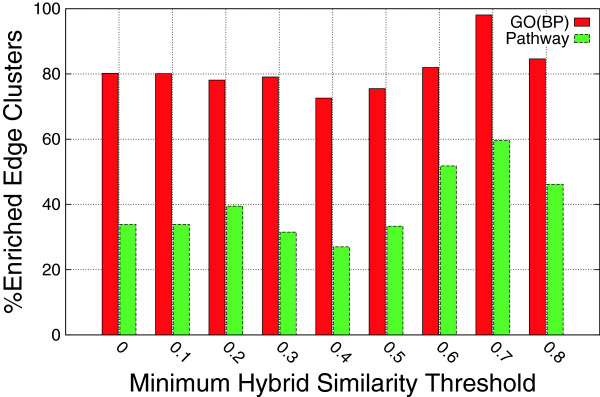
**Enrichment analysis.** Enrichment of functional annotations in the edge clusters for varying hybrid similarity thresholds. The Gene Ontology (GO) biological process term and the KEGG pathway databases were used for enrichment analysis.

**Figure 5:**
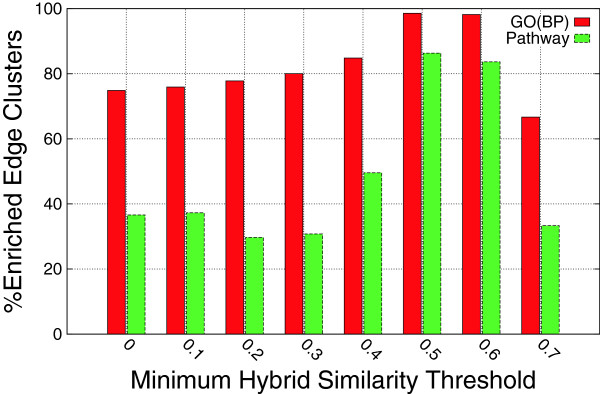
**Enrichment analysis.** Enrichment of functional annotations in the edge clusters for varying hybrid similarity thresholds (*α* = 0.5).

To assess the biological significance of the reported edge clusters, we performed functional enrichment analysis of Gene Ontology (GO) biological process terms as well as KEGG pathways. The enrichment analysis was performed using the DAVID tool [21,22]. In the analysis, only edge clusters with at least five genes were included. If the set of genes in an edge cluster is significantly enriched with at least one biological process GO term, then we say that the edge cluster is enriched. We computed the percentage of enriched edge clusters reported from several hybrid graphs for varying hybrid similarity thresholds. We have seen in the topological analysis of the reported edge clusters that for higher hybrid similarity thresholds, the reported edge clusters have higher recurrence. As shown in Figure 4, the percentage of enriched modules is higher for large hybrid similarity thresholds, thus for recurrent modules. Moreover, the same trend holds for KEGG pathways enrichment. The percentage of edges clusters that are enriched in KEGG pathways is much higher for larger *β* thresholds. The same trend is observed for different *α* values that control the contribution of the edge structural similarity. Figure 5 illustrates the percentage of enriched edge clusters *α* = 0.5. For *α* = 0.5 and *β* = 0.5, the algorithm reported 276 edge clusters, 139 of which had at least 5 genes. Some of the biological process GO terms that were highly enriched in these 139 modules include cell cycle phase,’ ‘cell cycle process,’ ‘cell cycle,’ ‘mitotic cell cycle,’ ‘M phase of mitotic cell cycle,’ and ‘cell proliferation.’ These results corroborate the premise of this research that integrative analysis of gene expression data reveal meaningful biological insights.

### Impact of edge pruning

As mentioned in the Methods section, we pruned infrequent edges that appear in less than 10 graph layers. When we decrease the edge pruning threshold, more edges qualify to be included in the summary graph and thus increasing the density of the summary graph. For example, for a frequency threshold of 5, the summary graph has 12,149 nodes and 1,785,621 edges (density =0.024). When we decreased the threshold to 3, the summary graph has a much larger number of edges (10,951,387) with 12,490 nodes (density = 0.14). Recall that the size of the final hybrid graph is much larger than that of the summary graph.

**Figure 6:**
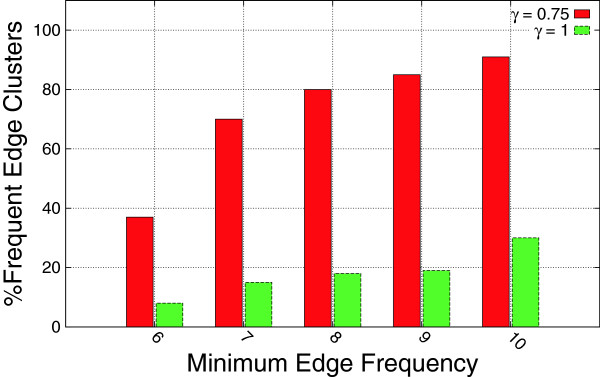
**Impact of edge pruning on the percentage of frequent edge clusters.** An edge cluster is frequent if it appears in at least 7 graphs. An *α* value of 0.5 and a *β* value 0.5 were used to generate the hybrid graph.

We have noticed that for a small edge frequency threshold (*k*), a large percentage of the reported edge clusters are not frequent in at least *k* coexpression networks. Figure 6 shows the effect of the edge frequency threshold on the percentage of frequent edge clusters. It is clear that as we increase the edge frequency threshold, the percentage of frequent edge clusters (frequent in at least 7 graphs) increases. As we shall see next, this has a direct impact on the running time of the algorithm.

### Recurrent edge clusters in random networks

To investigate how likely frequent patterns can be observed in randomly generated graphs, we generated 52 random coexpression networks with the same number of nodes, and edges as those observed in the actual 52 coexpression networks. We ran the proposed algorithm on this datasets and topologically analyzed the reported patterns in terms of recurrence. We observed that the size of the hybrid similarity graph was similar to that of the hybrid similarity graph constructed from the actual networks. However, the average value of the hybrid similarity was significantly less in the hybrid graph constructed from random networks.

For reasonable *β* threshold values (≥ 0.3), the randomized hybrid similarity graphs have a relatively small number of edges (hundreds) with hybrid similarities that exceed the threshold. Moreover, these similar edges are fragmented and do not belong to well-connected modules. We have noticed that the reported edge clusters from these small hybrid graphs are not frequent. For example, for a frequency threshold of 5, none of the reported edge clusters were frequent. In random networks, we can get edges that appear simultaneously but infrequently. These edges will have high attribute similarity and will be part of the final hybrid similarity graph. However, the edge clusters extracted from the hybrid graph are not frequent when the frequency threshold is set higher than the support of these edges.

### Comparison to NetsTensor

In order to get a sense of how the reported results compare with the modules reported by other approaches, we ran the NetsTensor algorithm [5] on the same dataset. The algorithm reports recurrent heavy modules. A set of genes is a recurrent heavy module if the set of genes meet a minimum density (heaviness) threshold in at least a minimum number of coexpression networks. For a minimum frequency threshold of 5, and a heaviness threshold of 0.5, the algorithm reported 12,763 recurrent heavy modules. The average size of these patterns is 5 genes and the average recurrence of these patterns is 9 graphs. A similar trend was observed for heaviness thresholds of 0.4,0.5,0.6, and 0.7. For a small heaviness threshold of 0.4, the average recurrence increased to 10, and for a large heaviness threshold of 0.7, the average recurrence dropped to 6 graphs. This can be due to the fact that the heaviness constraint is strict and not many modules will have its genes showing similar expression in a significant number of datasets.

Functional enrichment analysis of the reported modules (minimum recurrence of 5, and heaviness equals 0.5) revealed that about 50*%* of these modules are enriched with at least one biological process GO term. Moreover, 17*%* of the patterns are enriched with at least one KEGG pathway. In consistence with the findings reported in [5] on a different dataset, we observed that the reported modules of the NetsTensor approach have higher percentage of functionally enriched modules when we mine for heavier and more recurrent modules. It is important to note that the NetsTensor approach works on weighted and unweighted coexpression networks, and is able to extract more biologically-relevant patterns from weighted networks. However, since the focus of this work is on unweighted networks, we ran the NetsTensor approach on unweighted coexpression networks.

Our approach finds recurrent coexpression link modules regardless of the density of these modules. In the NetsTensor approach, the minimum recurrence and heaviness are two parameters that control the running time and the number of the reported patterns depend on these parameters. For recurrence and heaviness thresholds of 5 and 0.5, the algorithm took more than 19 hours. When we decreased the edge pruning threshold from 10 to 7 and ran the NetsTensor algorithm on the dataset of 9,784 genes, the algorithm did not finish in fifteen days. The authors of the NetsTensor algorithm reported that the algorithm took 200 hours (more than eight days) to finish on a dataset of 131 microarray datasets [5].

### Complexity and running time

There are two main tasks in the proposed algorithm: constructing the hybrid graph and clustering. The summary graph *G*_*S*_(*V*, *E*_*S*_) has |*V*| nodes and |*E*_*S*_| edges. The number of nodes in the topological similarity graph is |*E*_*S*_|. Since the edge attributed similarity is only calculated for edge pairs that are topologically similar, the hybrid graph has the same number of nodes and edges as the topological similarity graph. In the worst case, the summary graph will be a complete graph and thus the number of edges, |*E*_*S*_|, equals to |*V*|∗(|*V*|-1)/2 which is *O*(|*V*|^2^). Calculating the topological similarity matrix takes *O*(|*E*_*S*_|^2^|*V*|) (there are |*E*_*S*_|^2^ edge pairs) and calculating the attribute similarity takes *O*(|*E*_*S*_|^2^). Thus, constructing the hybrid similarity matrix takes *O*(|*E*_*S*_|^2^|*V*|) which is *O*(|*V*|^5^).

The worst case running time for the MCL algorithm is *O*(|*E*_*S*_|∗*K*^2^) for sparse graph, where *K* is a parameter that controls the maximum nonzero entries per stochastic column [15]. Therefore, the computational complexity of the proposed algorithm is *O*(|*E*_*S*_|^2^|*V*|) which is *O*(|*V*|^5^).

The running time of the algorithm depends on the size of the summary graph which is controlled by how much edge pruning we and the size of the hybrid graph that is mainly controlled by *β*. For the datasets, we have an edge frequency threshold of 10, the summary graph has 4,133 genes and 32,741 links. For *β*=0 (all hybrid similarities are retained), the hybrid graph has 2,273,510 edges, and the running time is about 1,218 seconds. For *β*=0.5, the hybrid graph has only 15,779 edges, and the algorithm took 89 seconds.

For both cases, the algorithm took 84 seconds to construct the hybrid graph, and the remaining time for clustering phase. We also observed that when topological similarity is considered in calculating the hybrid similarity (i.e., increase *α*), the hybrid graph gets smaller and thus the running time of the algorithm improves.

As we discussed earlier, pruning infrequent edges has an impact on the size of summary graph and thus the size of the final hybrid graph (depending on *β*) and the running time of the algorithm. As we decreased the edge pruning threshold from 10 to 7, the summary graph is much larger; 9,784 genes and 308,162 links. For an *β*=0, the algorithm took 21,360 seconds (six hours) to finish. By retaining edge similarities of at least 0.2 and 0.3, the algorithm took 628 and 95 seconds, respectively.

## Conclusion

We have proposed an algorithm for biological module mining from coexpression networks representing multiple gene expression datasets. This algorithm leverages the similarity of the occurrence of coexpression links to build a weighted hybrid similarity graph whose nodes represent the coexpression links and edges in the hybrid graphs capture how similar two links are in terms of their occurrence in the multiple graphs and their position in the graph structure. Biological modules are then discovered from the hybrid similarity network using graph clustering. Since we cluster edges, the proposed approach is able to discover overlapping modules. This enables the discovery of recurrent interaction patterns that are present in multiple coexpression networks.

Experimental results on 52 Human gene expression datasets show that proposed approach discovers biologically significant patterns. Functional enrichment analysis show that the reported modules are highly enriched with biological process GO terms and KEGG pathways. We have observed that the more recurrent the edge clusters are, the more functionally homogeneous they are. The occurrence of a set of coexpression links in multiple coexpression graphs corroborates their biological significance. Moreover, the presence of coexpression links in multiple datasets alleviates the problems associated with biological inference based on a single gene expression data. Our results indicate that integrative analysis of multiple gene expression data is essential and promising. This algorithm is applicable to any domain with multiple networks on the same set of nodes.

## Competing interests

The authors declare that they have no competing interests.

## Authors’ contributions

SS and CO discussed the idea. SS designed the algorithm and wrote the paper. SS and CO worked on experimental design and patterns topological analysis. Both authors read and approved the final manuscript.
